# Underreporting of diabetes mellitus as the cause of death in Bauru, State of São Paulo, Brazil over 40 years: a documental study

**DOI:** 10.20945/2359-4292-2023-0443

**Published:** 2024-06-24

**Authors:** Lucas Casagrande Passoni Lopes, Gabriel Araújo Medeiros, Mauro Wieczorek, Marina dos Santos de Carvalho Pinto, Carlos Antonio Negrato

**Affiliations:** 1 Faculdade de Odontologia de Bauru Universidade de São Paulo Bauru SP Brasil Faculdade de Odontologia de Bauru, Universidade de São Paulo, Bauru, SP, Brasil

**Keywords:** Diabetes, underreport, death certificate

## Abstract

**Objective:**

To evaluate, characterize and search for trends in the underreporting of diabetes mellitus (DM) as the cause of death in Bauru, São Paulo, Brazil, over 40 years.

**Subjects and methods:**

This was a documental study. Clinical and mortality data were collected from individuals known to have type 1 (DM1) and type 2 diabetes mellitus (DM2), residing in Bauru, State of São Paulo, followed at a local endocrinology clinic from 1982 to 2021, who deceased during this period.

**Results:**

A significant underreporting of DM as the cause of death (64.41%) was found, mostly associated with male gender (OR = 1.59 [95% CI: 1.18; 2.15]; p < 0.01), DM2 (OR = 2.64 [95% CI: 1.32; 5.26]; p < 0.01), dying in the first decade of the study (OR = 4.07 [95% CI: 1.54; 10.71]; p < 0.001) and shorter DM duration (OR = 1.02 [95% CI: 1.01; 1.04]; p < 0.01). Age, type of treatment, body mass index, marital status and ethnicity, did not show a significant association with DM underreporting. There was a decreasing trend in DM1 underreporting (Decade Percentual Change = -7.10 [95% CI: -11.35; -3.40]), but a stationary trend for DM and DM2. The main primary cause of death was cardiovascular-related complications.

**Conclusion:**

The underreporting of DM as the cause of death was very frequently found, and was associated with male gender, decade of death, shorter DM duration and DM2. If our data could be applied to the whole country, DM would possibly emerge as a more prominent cause of death in Brazil. Future studies in other cities and geographic regions are warranted to confirm our findings.

## INTRODUCTION

Diabetes mellitus (DM) is a chronic degenerative disease, which has a high and growing prevalence in the Brazilian and worldwide population. The two most prevalent types of DM are type 1 diabetes mellitus (DM1) and type 2 diabetes mellitus (DM2), which account for about 10% and nearly 90% of all cases, respectively (1). According to a Brazilian multicenter study conducted in the nineties, 7.6% of people aged between 30 and 69 years had the diagnosis of DM (2). Later surveys carried out in the cities of Ribeirão Preto and São Carlos, both located in the State of São Paulo, found that 12.1% and 13.5% of the evaluated people, in similar age groups, had DM, respectively (3,4). In these studies, no differences in prevalence were found regarding gender and ethnicity, in addition to the fact that about half of the people were unaware of their diagnosis (2,4). Among those who knew they had DM, about 25% did not receive any type of treatment (2). These data show that DM is a serious public health problem in Brazil, due to its high prevalence, wide range of serious related complications, and high costs involved in its treatment, which overload the public health system (5).

Death certificates (DC) are official documents with a prominent epidemiological role since, based on them, it is possible to perceive the pattern of diseases in each region, helping to support government actions aimed at promoting health and disease prevention, in addition to providing data that can be used as important indicators of health and socioeconomic conditions of the general population (6,7). However, these certificates are flawed concerning the quality of their completion, which may lead to underreporting the real causes of death due to several diseases, including DM, considered the 7th most prevalent cause of death among Brazilians (8).

This study aimed to evaluate the underreporting of DM as the cause of death in people with a confirmed diagnosis of DM who died within forty years (1982-2021) in the city of Bauru, State of São Paulo, Brazil.

## SUBJECTS AND METHODS

This was a documental, observational study with a quantitative approach. Clinical and mortality data were collected from individuals known to have DM1 and DM2, residing in Bauru, State of São Paulo, who were treated at a local endocrinology clinic over forty years (1982 to 2021) and deceased during this timeframe. This clinic serves patients from primary, secondary, and tertiary levels of care, all of them referred to be attended initially at Bauru’s Diabetics Association (an outpatient clinic specialized in DM that receives patients from all levels of care, living in Bauru), representing consequently, the whole population with DM living in town.

This study was approved by the Research Ethics Committee of the Faculty of Dentistry of Bauru, University of São Paulo, with the following number: 37022220.0.0000.5417. Judicial authorization was also obtained, allowing the analysis of data contained in all available DC.

The study followed the Strengthening the Reporting of Observational Studies in Epidemiology guidelines (9).

### DM underreporting

As mentioned in the instruction manual for filling DC of the Brazilian Ministry of Health, it would be expected that patients with DM should have this condition mentioned in part I of their DC, if DM is the direct cause of death or, at least, in part II, if DM is a comorbidity of this given patient. Thus, the underreporting of DM as the cause of death can be defined as the absence of this condition on the DC of a patient who had DM.

### Data collection

Data such as age, gender and self-reported color/ethnicity (White, Black, Brown, Yellow and Indigenous) according to the Brazilian Geography and Statistics Institute (IBGE) (10) were collected. For statistical analysis purposes, ethnicities were grouped as “Whites” and “non-Whites”. Data regarding marital status (single, married, widowed, and divorced), type of DM, DM duration (defined as the length of time between DM diagnosis and death, being randomly divided in ten years periods as follows: less than 10 years; between 10 and 19.9 years; 20 and 29.9 years; 30 and 39.9 years and finally, more than 40 years), modality of DM treatment (diet, oral antidiabetic agents, insulin, insulin plus oral antidiabetic agents), weight, height and body mass index (BMI), which was calculated dividing weight (kg) by height in square meters (m^2^), were collected. Patients were classified as being underweight if BMI < 18.5; with a normal weight if BMI was between 18.6 and 24.9; overweight if BMI was between 25 and 29.9; grade I obesity if BMI was between 30 and 34.9; grade II obesity if BMI was between 35 and 39.9; and grade III obesity if BMI was > 40 (11).

To be included in this study, patients had to be Bauru dwellers at the moment of their deaths, had to have received medical care at the previously mentioned endocrinology clinic, died between 1982 and 2021, had the diagnosis of DM according to current criteria of the Brazilian Diabetes Society (12) and the American Diabetes Association (13), or were in use of medications to treat hyperglycemia, and had to have their DC registered in one of the two Registry Offices existing in town. Patients who did not meet these criteria were excluded from the study.

**Figure 1:**
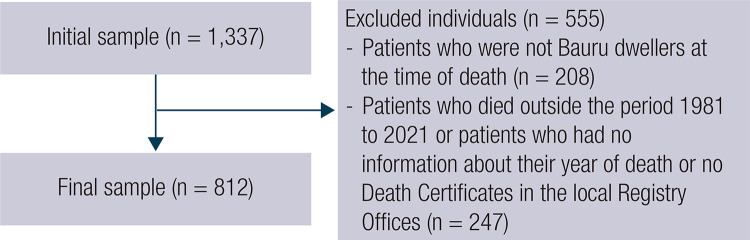
Flowchart of the selection of patients included in the final sample.

Data regarding the cause of death were obtained manually in the two Registry Offices responsible for DC in the municipality of Bauru. Data collection was assisted and supervised by a professional specifically designated by the respective Registry Office. A total of 913 certificates were retrieved, since some patients had already been excluded from the initial sample as depicted in Figure 1. Of these certificates, information regarding the cause of death of each patient was found in only 812 certificates. A possible explanation for the difference between the DC sought and those found by the Registry Offices, was that some individuals had the same names, which did not allow researchers distinguishing who they really were, without having additional information such as their parents’ names, in addition to the fact that many individuals, besides living in Bauru had their deaths registered in other localities. These data were transferred in an integral and equal manner to a Microsoft Excel^®^ spreadsheet and subsequently subjected to descriptive analysis.

### Logistic regression analysis

After an exploratory analysis, an association analysis was performed using binary Wald logistic regression, with DM reporting as the dependent (outcome) variable and as the independent (exposure) variables, we considered age, gender, self-reported color/ethnicity, marital status, type of DM, DM duration, modality of DM treatment, BMI and decade of death. Our sample satisfied the assumption of proportional odds, and no collinearity issues were detected among the variables. The goodness of fit was accessed through the Hosmer-Lemeshow test. Pearson’s Chi-square for association was also used to address differences between DM1 and DM2 groups. A *p*-value less than 0.05 was considered statistically significant. Forward and backward selections provided the final model with significant variables. The odds ratios (OR) were calculated with a 95% confidence interval (CI). All data were analyzed using the Statistical Package for the Social Sciences (SPSS version 25.0, SPSS, Inc., Chicago, Illinois, USA).

### Joinpoint regression analysis

Joinpoint regression analysis determined the magnitude of time trends in DM underreporting rates by calculating the decade percent change (DPC) and its 95% CI (14). The proportion variable was computed by using the number of non-reported deaths as the numerator and the total number of deaths as the denominator. This analysis was performed using the Joinpoint Regression Program (Version 5.0.2, Statistical Methodology and Applications Branch, Surveillance Research Program, National Cancer Institute).

## RESULTS

The initial sample was formed by 1,367 individuals, all of them diagnosed with DM and deceased between 1981 and 2021. From this sample, 555 individuals were excluded, 28 with DM1 and 527 with DM2, for not meeting all the inclusion criteria or for having any missing data; 812 individuals remained for analysis, 40 (4.93%) with DM1 and 772 (95.07%) with DM2, whose DC were assessed individually. Figure 1 describes the selection process of the included individuals.

Sociodemographic characteristics, clinical data, death according to decades and DM type, are shown in Tables 1 to 3.

Among the 812 certificates evaluated, DM was mentioned as the cause of death in 289 (35.59%), 23 (2.83%) patients with DM1 and 266 (32.76%) patients with DM2. At the same time, 523 (64.41%) certificates did not mention DM, 17 (2.09%) in patients with DM1 and 506 (62.32%) with DM2.

A logistic regression analysis of the underreporting of DM has found that males had increased odds of being underreported (OR = 1.592 [95% CI: 1.18; 2.15]) compared with females. Regarding DM duration, each one-year decrease in DM duration resulted in increased odds of being underreported (OR = 1.024 [95% CI: 1.01; 1.04]), which means that people who lived shorter periods with DM (less than ten years), were more likely to be underreported. BMI, ethnicity and treatment modality did not have any association with the underreporting of DM as the cause of death (p > 0.05) (Table 1 and Table 2).

**Table 1:** Sociodemographic characteristics of the studied population

	Total sum	Total	Total	DM1	DM1	DM2	DM2

		Notified	Not notified	Notified	Not notified	Notified	Not notified
Gender (Men)	370 (45.56%)	111 (13.66%)	259 (31.90%)	8 (0.98%)	7 (0.86%)	103 (12.68%)	252 (31.04%)
Gender (Women)	442 (54.44%)	178 (21.92%)	264 (32.52%)	15 (1.84%)	10 (1.23%)	163 (20.08%)	254 (31.29%)
Self-reported color/ethnicity (White)	717 (88.30%)	253 (31.16%)	464 (57.14%)	20 (2.46%)	15 (1.84%)	233 (28.70%)	449 (55.30%)
Self-reported color/ethnicity (Non-white)	95 (11.70%)	36 (4.44%)	59 (7.26%)	3 (0.37%)	2 (0.24%)	33 (4.07%)	57 (7.02%)
Age (Under 40 years)	21 (2.58%)	10 (1.23%)	11 (1.35%)	10 (1.23%)	7 (0.86%)	0 (0.00%)	4 (0.49%)
Age (Between 40 and 49 years)	30 (3.69%)	13 (1.60%)	17 (2.09%)	3 (0.37%)	5 (0.62%)	10 (1.23%)	12 (1.47%)
Age (Between 50 and 59 years)	81 (9.98%)	27 (3.32%)	54 (6.66%)	4 (0.49%)	1 (0.12%)	23 (2.83%)	53 (6.54%)
Age (Between 60 and 69 years)	172 (21.19%)	56 (6.90%)	116 (14.29%)	0 (0.00%)	1 (0.12%)	56 (6.90%)	115 (14.17%)
Age (Between 70 and 79 years)	234 (28.82%)	89 (10.96%)	145 (17.86%)	4 (0.49%)	2 (0.24%)	85 (10.47%)	143 (14.62%)
Age (Between 80 and 89 years)	205 (25.25%)	76 (9.35%)	129 (15.88%)	2 (0.24%)	0 (0.00%)	74 (9.11%)	129 (15.58%)
Age (Over 90 years)	69 (8.49%)	18 (2.21%)	51 (6.28%)	0 )0.00%)	1 (0.12%)	18 (2.21%)	50 (6.16%)
Marital status (Married)	585 (72.05%)	205 (25.26%)	380 (46.79%)	16 (1.98%)	12 (1.47%)	189 (23.28%)	368 (45.32%)
Marital status (Single)	74 (9.11%)	32 (3.94%)	42 (5.17%)	4 (0.49%)	3 (0.37%)	28 (3.45%)	39 (4.80%)
Marital status (Widower)	126 (15.52%)	44 (5.43%)	82 (10.09%)	0 (0.00%)	0 (0.00%)	44 (5.43%)	82 (10.09%)
Marital status (Divorced)	27 (3.32%)	8 (0.98%)	19 (2.34%)	3 (0.36%)	2 (0.24%)	5 (0.62%)	17 (2.10%)

Data presented as n (%); DM1 = Type 1 diabetes mellitus; DM2 = Type 2 diabetes mellitus

**Table 2:** Clinical data of the studied population

	Total sum	Total	Total	DM1	DM1	DM2	DM2

		Notified	Not notified	Notified	Not notified	Notified	Not notified
BMI (Underweight)	15 (1.84%)	4 (0.48%)	11 (1.36%)	2 (0.24%)	3 (0.37%)	2 (0.24%)	8 (0.99%)
BMI (Normal weight)	210 (25.88%)	74 (9.11%)	136 (16.37%)	19 (2.33%)	13 (1.61%)	55 (6.78%)	123 (15.16%)
BMI (Overweight)	297 (36.58%)	113 (13.92%)	184 (22.66%)	2 (0.24%)	1 (0.12%)	111 (13.68%)	183 (22.54%)
BMI (Grade I obesity)	193 (23.77%)	67 (8.25%)	126 (15.52%)	0 (0.00%)	0 (0.00%)	67 (8.25%)	126 (15.52%)
BMI (Grade II obesity)	59 (7.26%)	18 (2.21%)	41 (5.05%)	0 (0.00%)	0 (0.00%)	18 (2.21%)	41 (5.05%)
BMI (Grade III obesity)	38 (4.67%)	13 (1.60%)	25 (3.07%)	0 (0.00%)	0 (0.00%)	13 (1.60%)	25 (3.07%)
Treatment modality (Only diet)	40 (4.92%)	18 (2.21%)	22 (2.71%)	0 (0.00%)	0 (0.00%)	18 (2.21%)	22 (2.71%)
Treatment modality (Oral antidiabetics)	412 (50.74%)	132 (16.25%)	280 (34.49%)	0 (0.00%)	0 (0.00%)	132 (16.25%)	280 (34.49%)
Treatment modality (Insulin)	206 (25.38%)	76 (9.32%)	130 (16.01%)	23 (2.84%)	17 (2.09%)	53 (6.58%)	113 (13.92%)
Treatment modality (Oral antidiabetics plus insulin)	154 (18.96%)	63 (7.76%)	91 (11.20%)	0 (0.00%)	0 (0.00%)	63 (7.76%)	91 (11.20%)
Diabetes duration (Under 10 years)	125 (15.39%)	31 (3.81%)	94 (11.58%)	3 (0.36%)	2 (0.24%)	28 (3.45%)	92 (11.34%)
Diabetes duration (Between 10 and 19 years)	294 (36.21%)	87 (10.72%)	207 (25.49%)	6 (0.74%)	5 (0.61%)	81 (9.98%)	202 (24.88%)
Diabetes duration (Between 20 and 29 years)	278 (34.23%)	118 (14.53%)	160 (19.70%)	7 (0.86%)	5 (0.61%)	111 (13.67%)	155 (19.09%)
Diabetes duration (Between 30 and 39 years)	91 (11.21%)	45 (5.54%)	46 (5.67%)	4 (0.49%)	4 (0.49%)	41 (5.05%)	42 (5.18%)
Diabetes duration (More than 40)	24 (2.96%)	8 (0.98%)	16 (1.98%)	3 (0.36%)	1 (0.12%)	5 (0.62%)	15 (1.86%)

Data presented as n (%); BMI = body mass index; DM1 = type 1 diabetes mellitus; DM2 = type 2 diabetes mellitus.

**Figure 2:**
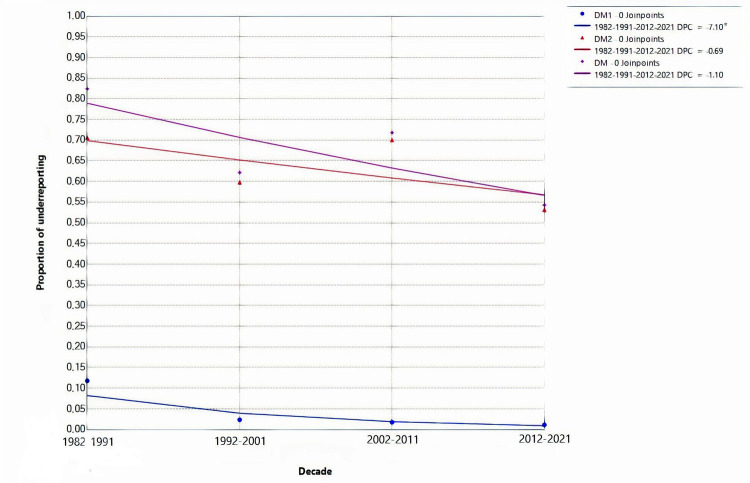
Reporting of diabetes as the cause of death according to diabetes type and decades.

Concerning DM type, patients who had DM2 presented higher underreporting rates compared with patients with DM1 (OR = 2.638 [95% CI: 1.32; 5.26]). Regarding temporal trends of underreporting, patients who deceased during the first decade (1982 - 1991) showed four times increased odds of being underreported (OR = 4.068 [95% CI: 1.54; 10.71]), compared with the last decade (2012 – 2021), which presented the lower proportion of underreporting. (Table 3). In time series analysis, DM1 underreporting had a decreasing trend, with a -7.10 DPC (95% CI: -11.35; -3.40), while DM and DM2 had stationary trends, as can be seen in Figure 2.

**Table 3:** Reporting of diabetes as the cause of death according to decades

Decade	Total sum	Total	Total	DM1	DM1	DM2	DM2

		Notified	Not notified	Notified	Not notified	Notified	Not notified
1982 to 1991	34 (4.19%)	6 (0.72%)	28 (3.47%)	2 (0.24%)	4 (0.48%)	4 (0.48%)	24 (2.99%)
1992 to 2001	169 (20.83%)	64 (7.89%)	105 (12.94%)	9 (1.11%)	4 (0.48%)	55 (6.78%)	101 (12.46%)
2002 to 2011	340 (41.84%)	96 (11.82%)	244 (30.04%)	6 (0.72%)	6 (0.72%)	90 (11.10%)	238 (29.32%)
2012 to 2021	269 (33.14%)	123 (15.14%)	146 (17.98%)	6 (0.72%)	3 (0.37%)	117 (14.42%)	143 (14.61%)

Data presented as n (%); DM1 = type 1 diabetes mellitus; DM2 = type 2 diabetes mellitus.

The final logistic regression model significantly improved the prediction of underreporting but explained only 9% (Nagelkerke) of a given patient being underreported as shown in Table 4.

**Table 4:** Final logistic regression model with DM underreporting as dependent variable

Variable categories	95% CI		

	OR	Lower	Higher
Male	1.59	1.18	2.15
Female	1		
Decades			
First decade (1982-1991)	4.07	1.54	10.71
Second decade (1992-2001)	1.25	0.82	1.91
Third decade (2002-2011)	2.03	1.44	2.88
Last decade (2012-2021)	1		
Diabetes duration	1.02	1.00	1.04
Type 2 diabetes	2.64	1.32	5.26
Type 1 diabetes	1		

CI = confidence interval; OR = odds ratio.

Among all evaluated DC, the main related causes of death were cardiovascular diseases, found in 167 (20.56%) individuals, followed by infectious diseases, found in 89 (10.96%); neoplasms, in 75 (9.23%); multiple organs failure, in 75 (9.23%); respiratory failure, in 73 (8.75%); acute and chronic renal disease, in 69 (8.28%); cardiorespiratory arrest, in 59 (7.07%); shock, in 35 (4.20%); systemic arterial hypertension in 31 (3.72%); polytrauma in 26 (3.12%); gastrointestinal diseases, in 25 (3.00%); Alzheimer’s, Parkinson and others dementias, in 22 (2.64%); sudden death in 18 (2.16%); liver failure in 16 (1.92%); acute abdomen in 15 (1.80%); hydroelectrolytic disorders, in 15 (1.80%); psychiatric disorders, in 12 (1.32%) and homicide, in 2 (0.24%) patients, respectively.

## DISCUSSION

In our sample, high rates (64.41%) of DM underreporting as the cause of death were found, mostly among patients who had DM2, males, with shorter DM duration and those who died in the first decade of the study. Age, treatment modality, BMI, marital status and ethnicity were not associated with underreporting. Cardiovascular complications were the most frequent cause of death found among these patients.

The higher rates of DM2 underreporting as the cause of death (66.38%) compared with those of DM1 (40%), could be explained by the higher prevalence of DM2 in the studied population (1), which could increase the chances of underreporting in this specific group.

Regarding gender, the higher rates of underreporting among males are in accordance with the findings of Andersson and Svärdsudd (1993) and Balkau and Papoz (1992), who found similar data in Scandinavia and France, respectively (15,16). However, these results disagree with the findings of previous studies carried out in Japan (17), England and Wales (18), which found higher rates among women. According to Silva and cols. (2022), the frequency related to gender in underreporting diseases such as DM is due not only to gender factors but also to sociocultural expressions that vary between individuals and different societies (19).

Our data have shown that underreporting of DM was associated with shorter DM duration, in accordance with Will and cols. (2001) (20), but in disagreement with Andersson and Svärdsudd (1993) for whom DM duration did not present an association with the underreporting of the disease as the cause of death (15). No hypothesis was suggested to explain this finding.

The highest DM underreporting rates in our study were observed between 1982 and 1991, with a decreasing trend for DM1, and a stationary trend for DM and DM2. At the same time, Will and cols. (2001) demonstrated a peak in DM underreporting between 1986 and 1993 and a decrease in underreporting of DM over the years (20), which was confirmed by Cheng and cols. (2008), who suggested there would be a better awareness of this disease in recent years, that could explain the existence of DM underreporting peaks in past decades and a trend in its reduction more recently (21). A possible explanation for this data variability could also be the locoregional differences of each study. At the same time, other diseases such as COVID-19, for example, especially in the pandemic period, might have influenced this variability, in some places. In fact, in 2020, a 19% excess of deaths occurred in Brazil due to COVID-19 (22). In the United States, in the same year, for every 120 deaths from general causes, there were 100 other deaths related to this viral disease (23). Thus, a possible reason for a higher or lower notification of DM is the increased notification of other diseases.

Our study did not find an association between age and underreporting of DM. However, several studies have found an association of underreporting of DM as the cause of death among older patients. In fact, Fuller and cols. (1983) and de Balkau and Papoz (1992) have shown that underreporting of DM increased as the patients’ age increased (16,18). Chen and cols. (2004) found a greater underreporting of DM in patients older than 60 years and Whittal and cols. (1990) found higher rates of underreporting in patients aged between 80 and 89 years (24,25). According to Silva and cols. (2022), the increase in underreporting of DM with increasing age could be related to the increased coexistence of DM with other comorbidities, such as obesity and cardiovascular diseases, which could cause a bias in reporting DM as the cause of death (19).

No association between treatment modality and the underreporting of DM was found in our study. However, Chen and cols. (2004) and Andersson and Svärdsudd (1993), have found a relationship between the use of insulin and lower rates of DM underreporting, which could be explained by the fact that insulin treatment could be a positive reinforcement for physicians remembering the diagnosis of DM when notifying deaths (15,24).

No relationship between BMI and underreporting of DM was found in this study. However, Stokes and Preston (2011), have found lower underreporting rates of DM as the cause of death, in patients with BMI > 30; however, these authors did not find a plausible explanation for this finding (26). According to Kos, a higher BMI in patients with DM could lead to underreporting of DM as the cause of death, since these patients present high mortality rates due to cardiovascular diseases, whose notification overlaps that of DM (27).

We did not find a relationship between DM underreporting and marital status. Nonetheless, Andersen and cols. (1993) observed that DM was underreported in about 60% of married patients and in 55% of unmarried patients (28). Indeed, according to Will and cols., a possible explanation for this finding is that spouses could be worse informants of the deceased partner’s health conditions than parents and offspring (20).

Ethnicity did not show an association with DM underreporting in DC in our study. Andersen and cols. (1993) did not find significant differences in DM underreporting between different ethnic groups (28). However, the underreporting of DM was higher in White individuals in the study performed by Stokes and Preston (2011), but opposite to the findings of Centers for Disease Control (CDC) (1991) according to which there was greater underreporting among Blacks (26,29). A possible explanation could be the ethnic frequencies of each location, and how people self-report their skin color.

Cardiovascular diseases were the main primary cause of death in our sample, as usually occur in patients with DM (30), which could play an important role in the underreporting of DM as the cause of death. Indeed, Andersson and Svärdsudd (1993) and Fuller and cols. (1983) found a higher underreporting rate of DM in conjunction with a higher prevalence of cardiovascular diseases (15,18). A possible explanation for this is that cardiovascular disease-related deaths tend to be more valued and, therefore, more notified than DM or other conditions (15). In parallel, the second cause of death in our sample was infectious diseases. In fact, patients with DM are more likely to develop infections since DM causes immune changes and promotes a constant inflammatory state that makes patients more susceptible to infections with worse prognosis (31). Consequently, an overlap between infectious and chronic diseases, such as DM, may occur, being infectious diseases much more reported, contributing consequently to the underreporting of DM as the cause of death (32). An important percentage of patients from this sample died due to neoplasms (9.23%), which are frequently related to DM, since these two share similar risk factors and common immune-endocrine alterations, which can also lead to overlapping between the two conditions and a subsequent underreporting of DM as the cause of death (33). Furthermore, a myriad of other causes were less frequently found but could have contributed to the underreporting of DM by being socially more impactful than DM (34).

Several studies discuss the real role of DM as the cause of death. Considering that DM is usually underreported as the cause of death, if correctly reported, its epidemiological impact would be much greater. Indeed, Balkau and Papoz suggest quadrupling the prevalence of DM (16), while Sasaki and cols. propose multiplying it by 6.42 times to reach real values (17). Taking into account the results of this study, we suppose that DM represents a more relevant cause of death than indicated by the Ministry of Health data (8). However, considering the lack of data of other possible causes of death, it is not possible to specifically determine the magnitude of this problem.

Will and cols. (2001) have proposed that there could be an improvement in the underreporting of DM as the cause of death over time, by improving screening strategies and giving greater assistance to patients with this condition. They have also suggested that better training of medical students and junior doctors, as well as better referencing of information between hospitals and DC centers, could improve this scenario (20).

Indeed, Fonseca and cols. have stated in the early seventies, that the quality of DC in Brazil was not satisfactory (35). Some technical manuals regarding the completion of DC were created by the Federal Council of Medicine and the Ministry of Health (36-38), online platforms training on how to fill these certificates (39) and even public policies such as the Data Initiative for Health (40) have also been adopted, trying to overcome this issue. All these initiatives point that the ideal DC should present in a logical and orderly manner, the initial condition that generated the entire succession of events that led to death. However, recent studies indicate that many DCs are still filled out inappropriately, generating confusing data with poor epidemiological relevance. This could be due to the lack of preparation of doctors on how to fill DCs which represents an important gap in current medical education (41). A study conducted in 2010 revealed that 20% of the evaluated doctors had never been instructed on how to complete a DC, and nearly 75% of them were unfamiliar with the Ministry of Health’s Instruction Manual for proper DC completion (42). Another possible explanation is that many professionals think that DC is just a bureaucratic formality, underestimating its fundamental epidemiological purpose and importance. It should be also mentioned that many doctors have no previous contact with the patient and/or their families being unaware that the patient had DM (28,35).

The strength of this study is that it was conducted with patients correctly diagnosed with DM, followed at the same endocrinology clinic, and death-related data were collected in the only two DC Registry Offices existing in town, so no case was lost. This study has also some limitations that must be addressed such as being conducted in only one center, the relatively small sample size, the use of convenience sampling, and the fact that some variables were grouped into categories for analysis, though reducing individual data discrimination. The present study may not be generalizable to other populations, and a more extensive time series analysis may be a good choice for further studies in other Brazilian cities and geographic regions, to accurately obtain the magnitude of DM underreporting in Brazil.

In conclusions, the underreporting of DM as the cause of death was very frequently found, and was associated with male gender, decade of death, shorter DM duration and DM2, while it was not associated with age, type of treatment, BMI, marital status and ethnicity. If our data should be applied to the whole country, it is possible that DM would emerge as a more prominent cause of death in our population. Future studies in other Brazilian locations and geographic regions are warranted to confirm our findings.

## References

[1] Brazilian Society of Diabetes (2023). https://diabetes.org.br.

[2] Malerbi DA, Franco LJ, The Brazilian Cooperative Group on the Study of Diabetes Prevalence (1992). Multicenter study of the prevalence of diabetes mellitus and impaired glucose tolerance in the urban Brazilian population aged 30-69 yr. Diabetes Care.

[3] Torquato MT, Montenegro RM, Viana LA, Souza RA, Lanna CM, Lucas JCB (2003). Prevalence study of diabetes mellitus and glucose intolerance in the urban population, aged 30 to 69 years, in the city of Ribeirão Preto - SP. Arq Bras Endocrinol Metab.

[4] Bosi PL, Carvalho AM, Contrera D, Casale G, Pereira MA, Gronner MF (2009). Prevalence of diabetes mellitus and impaired glucose tolerance in the urban population aged 30 to 79 years in the city of São Carlos, São Paulo. Arq Bras Endocrinol Metabol.

[5] Muzy J, Campos MR, Emmerick I, Silva RS, Schramm JM (2021). Prevalence of diabetes mellitus and its complications and characterization of gaps in health care based on research triangulation. Cad Saude Publica.

[6] Saito CK, Foloni AR, Oliveira CH, Tessarolli CF, Silva LM, Andrade A (2020). Analysis of filling out death certificates in Catanduva, São Paulo. Bioethics Magazine.

[7] Marinho MF, França EB, Teixeira RA, Ishitani LH, Cunha CC, Santos MR (2019). Data for health: impact on improving the quality of information on causes of death in Brazil. Rev Bras Epidemiol.

[8] Health Brazil Panels, general mortality - Causes of death, Saúde Brasil - Monitoring Panels, Content Centers, DAENT, SVS/MS Gov.br.

[9] Von Elm E, Altman DG, Egger M, Pocock SJ, Gøtzsche PC, Vandenbroucke JP (2007). The Strengthening the Reporting of Observational Studies in Epidemiology (STROBE) statement: guidelines for reporting observational studies. Lancet.

[10] IBGE, IBGE (2011). Sidra: IBGE automatic recovery system.

[11] World Health Organization (2000). Obesity: preventing and managing the global epidemic. Report of a World Health Organization Consultation.

[12] Cobas R, Rodacki M, Giacaglia L, Calliari LE, Noronha RM, Valerio C (2022). Official Guideline of the Brazilian Society of Diabetes. Connecting people.

[13] ElSayed NA, Aleppo G, Aroda VR, Bannuru RR, Brown FM, Bruemmer D (2023). on behalf of the American Diabetes Association. 2. Classification and diagnosis of diabetes: Standards of care in diabetes-2023. Diabetes Care.

[14] Kim HJ, Fay MP, Feuer EJ, Midthune DN (2000). Permutation tests for joinpoint regression with applications to cancer rates. Stat Med.

[15] Andersson DK, Svärdsudd K (1993). The value of death certification statistics in measuring mortality in persons with diabetes. Scand J Prim Health Care.

[16] Balkau B, Papoz L (1992). Certification of cause of death in French diabetic patients. J Epidemiol Community Health.

[17] Sasaki A, Horiuchi N, Hasegawa K, Uehara M (1993). The proportion of death certificates of diabetic patients that mentioned diabetes in Osaka, Japan. Diabetes Res Clin Pract.

[18] Fuller JH, Elford J, Goldblatt P, Adelstein AM (1983). Diabetes mortality: new light on an underestimated public health problem. Diabetologia.

[19] Silva FKS da, Ibiapina AB, Holanda EC, Batista CL, Silva JS da, Oliveira EH de (2022). Mortalidade por diabetes mellitus no estado do Piauí entre 2009 a 2019. Res Soc Develop.

[20] Will JC, Vinicor F, Stevenson J (2001). Recording of diabetes on death certificates. J Clin Epidemiol.

[21] Cheng WS, Wingard DL, Kritz-Silverstein D, Barret-Connor E (2008). Sensitivity and Specificity of Death Certificates for Diabetes: As Good as it Gets?. Diabetes Care.

[22] Guimarães RM, Oliveira MP, Dutra VG (2022). Excess mortality by cause group in the first year of the COVID-19 pandemic in Brazil. Rev Bras Epidemiol.

[23] Stokes AC, Lundberg DJ, Elo IT, Hempstead K, Bor J, Preston SH (2021). COVID-19 and excess mortality in the United States: a county-level analysis. PLoS Med.

[24] Chen F, Florkowski CM, Dever M, Beaven DW (2004). Death Certification and New Zealand Health Information Service (NZHIS) statistics for diabetes mellitus: an under-recognised health problem. Diabetes Res Clin Pract.

[25] Whittall DE, Glatthaar C, Knuiman MW, Welborn TA (1990). Deaths from diabetes are under-reported in national mortality statistics. Med J Aust.

[26] Stokes A, Preston SH (2011). Deaths attributable to diabetes in the United States: Comparison of data sources and estimation approaches. PloS One.

[27] Kos K (2020). Cardiometabolic Morbidity and Mortality with Smoking Cessation, Review of Recommendations for People with Diabetes and Obesity. Curr Diab Rep.

[28] Andersen EM, Lee JAH, Pecocaro RE, Koepsell TD, Hallstrom AP, Siscovick DS (1993). Underreporting of Diabetes on Death Certificates, King County, Washington. Am J Public Health.

[29] Centers for Disease Control (CDC) (1991). Sensitivity of death certificate data for monitoring diabetes mellitus-diabetic disease follow-up study.1985-1990. MMWR.

[30] Rodrigues TC, Canani LH, Gross JL (2010). Metabolic syndrome, insulin resistance and cardiovascular disease in type 1 diabetes mellitus. Arq Bras Cardiol.

[31] Moutschen MP, Scheen AJ, Lefebvre PJ (1992). Impaired immune responses in diabetes mellitus: Analysis of the factors and mechanisms involved. Relevance to the increased susceptibility of diabetic patients to specific infections. Diabetes Metab.

[32] Oliveira FA, Reis MA, Castro ECC, Cunha SFC, Teixeira VPA (2004). Doenças infecciosas como causas de morte em idosos autopsiados. Rev Soc Bras Med Trop.

[33] Shi Y, Hu FB (2014). The global implications of diabetes and cancer. Lancet.

[34] Franco LJ, Mameri C, Pagliaro H, Iochida LC, Goldenberg P (1998). Diabetes como causa básica ou associada de morte no Estado de São Paulo, Brazil, 1992. Rev Saúde Pública.

[35] Fonseca LAM, Laurenti R (1974). Qualidade da certificação médica da causa de morte em São Paulo, Brasil. Rev Saude Publica.

[36] Ministério da Saúde, Fundação Nacional de Saúde (2001). Manual de instrução para o preenchimento da declaração de óbito.

[37] Ministério da Saúde, Conselho Federal de Medicina, Centro Brasileiro de Classificação de Doenças (2009). A declaração de óbito: documento necessário e importante.

[38] Ministério da Saúde, Secretaria de Vigilância em Saúde, Departamento de Análise de Saúde e Vigilância de Doenças não Transmissíveis (2022). Declaração de Óbito: manual de instruções para preenchimento [recurso eletrônico].

[39] Ministério da Saúde, Secretaria de Vigilância em Saúde, Departamento de Análise de Saúde e Vigilância de Doenças não Transmissíveis Exercitando o preenchimento do Atestado das Causas de Morte.

[40] Bloomberg Philanthropies Data for health.

[41] Marinho MF, França EB, Teixeira RA, Ishitani LH, Cunha CC, Santos MR (2019). Dados para a saúde: impacto na melhoria da qualidade da informação sobre causas de óbito no Brasil. Rev Bras Epidemiol.

[42] Queiroz RC (2002). Validade e confiabilidade das declarações de óbito por câncer de boca no município do Rio de Janeiro.

